# Bilateral Anterior and Intermediate Uveitis with Occlusive Vasculitis as Sole Manifestation of Relapse in Multiple Sclerosis

**DOI:** 10.1155/2019/8239205

**Published:** 2019-07-22

**Authors:** Angelica Pedraza-Concha, Karin Brandauer, Alejandro Tello, Carlos Mario Rangel, Christian Scheib

**Affiliations:** ^1^Faculty of Medicine, Universidad Autónoma de Bucaramanga, Bucaramanga 680003, Colombia; ^2^Augenklinik am Diakonissenkrankenhaus, Karlsruhe 76199, Germany; ^3^Cornea and Anterior Segment Division, Centro Oftalmológico Virgilio Galvis, Bucaramanga 680003, Colombia; ^4^Retina and Posterior Segment Division, Centro Oftalmológico Virgilio Galvis, Bucaramanga 680003, Colombia

## Abstract

76-year-old female patient, with past medical history of relapsing-remitting multiple sclerosis manifested by retrobulbar optic neuritis in both eyes with an interval of one year between the first episode in the left eye and the one in the right eye and after three decades of remission, who consulted due to bilateral blurred and foggy vision. Subsequently, several differential diagnoses where ruled out. Diagnosis of bilateral anterior and intermediate uveitis with occlusive vasculitis attributed to a new relapse episode of multiple sclerosis was made, as the association between multiple sclerosis and intermediate uveitis is known, though the causal association is still questioned. This case shows how multiple sclerosis may only manifest with ocular involvement and exemplifies the broad spectrum of manifestations and complications, taking into account that ischemic areas from vasculitis and other comorbidities led to macular edema and unfortunately, prognosis became poorer. The complex course of the case enables emphasizing the responsibility role of the ophthalmologists in such systemic entities that may compromise the eye, in which suspicion of the disease and an adequate timing management approach is essential.

## 1. Introduction

The prevalence of multiple sclerosis (MS) in patients with any type of uveitis has been reported between 0,9 and 1,7%. On the other hand, prevalence of uveitis among patients with MS varies in a wide range though reaching very high frequency in some studies (between 0,65 and 36,7%) [1, 2]. Although causal association is still questioned, since the uvea has no myelin, an explanation of the relationship is their partially common embryonic neuroectodermic origin and association with HLA DR15 [2]. Other posterior segment condition, namely, retinal vasculitis, has been found between 10% and 35% of patients with MS, but retinal neovascularization, on the other hand, is an uncommon complication [3–5].

Furthermore, macular edema can complicate uveitis, in which most important risk factors are the severity and chronicity of the disease, aging, and smoking. The risk to develop cystoid macular edema (CME) related with intermediate uveitis is fourfold increased when the patient is a smoker [6].

Herein, we report the complex course of a patient with relapsing-remitting MS history, who was asymptomatic without receiving any treatment for more than 30 years and who presented a recurrence of the condition that manifested only with visual symptoms and ocular findings, only this time without affecting the optic nerve; consequently, turning out to be a diagnostic and management challenge.

## 2. Case Report

A 76-year-old, German, Caucasian female, with past medical history of relapsing-remitting MS (in remission for more than three decades), chronic obstructive pulmonary disease, hypertension, and heavy cigarette smoker, consulted to the emergency department in 2016 referring increased glare and sudden fall of visual acuity (VA) on both eyes (OU) since a couple of days. Ophthalmological background was left eye (LE) amblyopia and retrobulbar optic neuritis in the LE in 1986 and in the right eye (RE) in 1987, which along with the findings in Magnetic Resonance Imaging (MRI) of the brain led to the diagnosis of MS. Both episodes were treated with Azathioprine but, since then, did not receive additional therapy. The patient denied additional neurological symptoms for the last 30 years. She had cataract surgery with intraocular lens (IOL) implantation in OU in 2012. She noted decreased vision in the RE around six months before consulting in 2016, and diagnoses of anterior uveitis and vitreous hemorrhage (VH) in the right eye (RE) were done elsewhere. However, the patient did not continue to attend the treating ophthalmologist and thus did not receive specific management. She consulted the emergency department two days after noticing a sudden and greater decrease in VA in OU.

Examination approach: VA in the RE was Counting Fingers and 0.32 in the LE (Snellen decimal scale), which did not improve with spectacles. Intraocular pressure (IOP) was 19 mmHg in the RE and 22 mmHg in the LE. Slit lamp examination revealed in OU marked inflammation in anterior chamber (cells 3+) and granulomatous endothelial precipitates. In addition, posterior synechia and precipitates on the IOL front surface were observed in the LE. Vitreous turbidity, vitreous cells and snowballs, and retinal vessels with mild sheathing and adjacent hemorrhages were found in OU.

**Figure 1:**
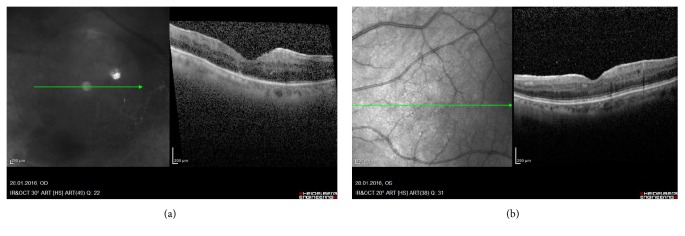
(a) RE, inflammatory cells in vitreous humor, epiretinal membrane, preserved foveal architecture with parafoveal retinal thickening (mild macular edema), without cysts or serous detachment (b) LE, inflammatory cells in vitreous humor, epiretinal membrane without traction, preserved foveal architecture without cysts or serous detachment.

**Figure 2:**
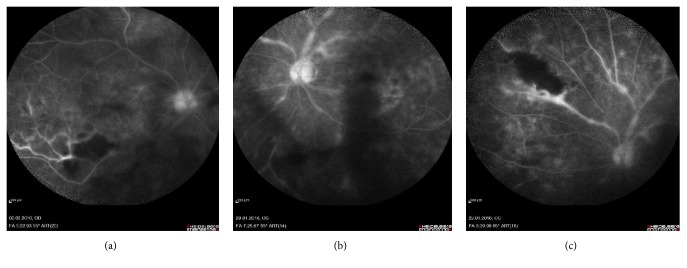
(a) RE, blockage of choroidal fluorescence secondary to vitreous opacities, pronounced ischemic areas in inferotemporal arcade, collateral vessel formation, and leakage near the optic nerve head and along the retinal veins. (b,c) LE, blockage of choroidal fluorescence secondary to vitreous opacities, leakage in foveal area along the retinal vessels, and staining of nasal retinal veins.

B-scan Macular Optical Coherence Tomography (OCT) from the RE revealed inflammatory cells in vitreous humor, epiretinal membrane, and preserved foveal architecture, with parafoveal retinal thickening (mild macular edema), without cysts or serous detachment (Figure 1(a)). In the LE, inflammatory cells in vitreous humor, epiretinal membrane without traction, and preserved foveal architecture without cysts or serous detachment were revealed (Figure 1(b)). Fluorescein angiography showed on the RE blockage of choroidal fluorescence secondary to vitreous opacities, pronounced ischemic areas in inferotemporal arcade, collateral vessel formation, and leakage near the optic nerve head and along the retinal veins (periphlebitis) (Figure 2(a)). In the LE, blockage of choroidal fluorescence secondary to vitreous opacities, leakage in foveal area (macular edema (ME)) and along the retinal vessels, and staining of nasal retinal veins (periphlebitis) were shown (Figures 2(b) and 2(c)).

Neurological approach proved intact cranial nerves function, no paresis, no pyramidal tract signs, and adequate reflexes. MRI of the orbit exhibited no evidence of retrobulbar optic neuritis. Skull MRI revealed no evidence of acute inflammatory cerebral foci.

Paraclinical tests helped ruling out differential diagnosis. Sarcoidosis was excluded by normal serum angiotensin converting enzyme, and there were no relevant abnormalities on chest x-ray. Other diseases possibly associated with ocular signs were not likely on absence of systemic clinical findings, negative ANA, and ANCA, normal HbA1c (5,2%), and no heart rhythm abnormalities. Infectious etiologies such Syphilis, Bartonellosis, and Lyme disease were also ruled out. Regarding ischemic causes, Carotid Doppler showed no plaques, dissection, or stenosis. Furthermore, the absence of malignant suspicious lymphocytes (dysplasic, pleomorphic lymphocytes with elevated nucleus-cytoplasm ratio) in a vitreous sample from RE excluded ocular lymphoma (Masquerade syndrome). PCR tests in that sample were negative for Herpes simplex, Varicella Zoster, Cytomegalovirus, and Mycobacterium Tuberculosis. Eventually, given the exclusion of other possible etiologies, diagnosis was a bilateral anterior uveitis and intermediate uveitis with occlusive vasculitis and mild macular edema, attributable to a MS relapse.

Intravenous methylprednisolone, 250 mg q.i.d. for 5 days, was administered. Topical prednisolone was indicated, initially every hour, and then tapered gradually over around two months. Cyclopentolate b.i.d. was used for several weeks. This scheme led to improvement of the findings, but Dorzolamide/Timolol b.i.d. and Brimonidine t.i.d. had to be used to control IOP increase.

At discharge, 5 days after the admission, VA from the RE was 0.1 and 0.32 on the LE, which did not improve with optical correction. IOP was normal in OU. Oral steroid taper schedule and hypotensive eye drops were prescribed. On the third month's control, even under daily oral steroids, bilateral vasculitis persisted. Consequently, interferon beta-1a (Rebif®) was initiated (44 mcg, 3 times per week, subcutaneously). On the sixth month's visit, CME was found in OU, despite the fact that she was receiving interferon beta-1a and 7.5 mg oral prednisolone. Bilateral subtenon injections of triamcinolone were performed. Two months later, CME persisted and a dexamethasone intravitreal implant (Ozurdex®) was injected in the RE and three months later in the LE as well.

Due to side effects with interferon beta-1a (Flu-like symptoms and depression), eight months after it was started, she was switched to peginterferon beta1-a (Plegridy®) 125 mcg injected subcutaneously every 2 weeks. A stable response to treatment was achieved, without inflammatory or neovascularization signs and improvement of CME for a couple of months. Nevertheless, due to persistence of ischemic findings in the retina (ischemic areas, collateral vessel formation, and mild macular edema reappearance) evidenced in fluorescein angiography image control, which possibly triggered recurrence of macular edema, despite having an adequate control of the inflammatory component, pan-retinal photocoagulation was performed in OU, in three sessions between 7 and 8 months after the initial consult, with satisfactory evolution. CDVA at the last check-up visit was 0.4 in the RE and 0.5 in the LE.

## 3. Discussion

In this patient with past medical history of relapsing-remitting MS, other causes of bilateral anterior and intermediate uveitis were ruled out, including viral infections (Herpes simplex, Varicella Zoster, and Cytomegalovirus), Sarcoidosis, and Systemic Lupus Erythematosus. Since vasculitis was present, further causes were also considered: Toxoplasmosis, Tuberculosis, Syphilis, and Borreliosis, but the results were also negative [1, 2, 5, 7].

Retinal vasculitis in MS occurs in a range of about 9-23% of the patients, and is almost exclusively limited to the veins [5]. Though rare, there are also cases in which MS presents only with retinal vessel affection and not even uveitis nor optical neuritis are present [5].

Intermediate uveitis itself has an overall favorable prognosis on absence of complications [2]; however when retinal vasculitis is present the possibility of developing CME and the risk of vision loss rises. Vasculitis may lead to areas of ischemia. Macular ischemia increases 4.4 times the risk of visual loss, and ischemia concerning ≥2 quadrants raises risks of neovascularization [4, 5]. Also, periphlebitis increases the risk of new relapsing events in the following 2 years [4, 7].

As for treatment approach, some authors have hypothesized that interferon beta might slow down the course of intermediate uveitis related to MS [8–10]. In the case we reported due to persistent bilateral vasculitis, interferon beta-1a was initiated 3 months after starting systemic and topical steroids. However bilateral CME developed and the patient initially required bilateral triamcinolone subtenon injection and then injection of dexamethasone intravitreal implant (Ozurdex®) in OU. Finally, due to persistent findings of retinal ischemia pan-retinal laser photocoagulation was performed in OU. The patient did not develop further complications of retinal ischemia, and in her nonamblyopic right eye the CDVA was preserved (0.4 in Snellen decimal scale) after the laser procedure.

Regarding diagnostic procedures, vitreous biopsy is not free of risks, and its use should be considered only when there is a significant doubt on the cause of the uveitis affecting the vitreous, especially when malignancy needs to be ruled out [11]. In this case, a vitreous biopsy performed in the RE using a 21G needle on a 1 ml syringe and was useful to rule out lymphoma (the immunophenotyping of the cells in the vitreous aspirate showed no evaluable lymphocyte population giving no indication of proliferation of lymphocytes). In addition, PCR results were negative for Herpes simplex, Varicella Zoster, Cytomegalovirus, and Mycobacterium Tuberculosis.

The VH initially found by an ophthalmologist elsewhere in the RE may be explained mainly due to retinal neovascularization secondary to the retinal ischemia, which in fact persisted in spite of dexamethasone intravitreal implant (Ozurdex®) and interferon, and pan-retinal photocoagulation was eventually required in OU.

This case exemplifies the broad spectrum of ocular manifestations and complications of MS. Given the background, ophthalmologists have great responsibility on suspicion of systemic diseases, such MS, when facing a patient with uveitis, since early diagnosis, therapy, and close follow-up play a significant role in prognosis [2, 12].
